# Disappearance of white sharks leads to the novel emergence of an allopatric apex predator, the sevengill shark

**DOI:** 10.1038/s41598-018-37576-6

**Published:** 2019-02-13

**Authors:** Neil Hammerschlag, Lacey Williams, Monique Fallows, Chris Fallows

**Affiliations:** 1 Rosenstiel School of Marine and Atmospheric Science, Department of Marine Ecosystems and Society, University of Miami, Miami, FL USA; 2 Leonard and Jayne Abess Center for Ecosystem Science and Policy, University of Miami, Coral Gables, Coral Gables, FL USA; 3 Apex Shark Expeditions, Shop 3 Quayside center, Simonstown, Cape Town, 7975 South Africa

## Abstract

Despite global declines of apex predatory sharks, evidence for ecosystem consequences remains limited and debated. This is likely a result of both the logistical difficulties of measuring such processes in marine systems and also due to shifting baselines, whereby the ecosystem changes have occurred prior to monitoring. Between 2000–2018, we conducted standardized monitoring of white shark (*Carcharodon carcharias*) abundance patterns (N = 6,333 shark sightings) and predatory activity (N = 8,076 attacks on seals) at Seal Island, a Cape fur seal (*Arctocephalus pusillus pusillus*) colony in False Bay, South Africa. Over the 18-year study, declines in white shark abundance and attack rates were documented between 2015–2018, with anomalous lows occurring in 2017 and 2018. This included prolonged periods of complete white shark absence from Seal Island. The disappearance of white sharks from Seal Island coincided with the unprecedented appearance of sevengill sharks (*Notorynchus cepedianus*; N = 120 sightings), an otherwise allopatric kelp-associated apex predator in False Bay. We also recorded a sevengill shark attacking a live seal in the absence of white sharks. These data provide empirical evidence for behavioral shifts in an allopatric marine predator following the decline and disappearance of white sharks from a foraging site. This study demonstrates the importance of historical data and long-term monitoring for disentangling ecological consequences of apex predator declines.

## Introduction

It has been suggested that the loss of apex predators is humankind’s most pervasive influence on the natural world, with far reaching ecological effects via trophic downgrading^1^. Exploitation and subsequent declines in large (>2 m) sharks has occurred in oceans globally, driven largely by overfishing for their fins and meat^2^. Accordingly, the ecosystem consequences of such declines are an area of great concern. However, evidence for such ecological consequences are both limited and an area of debate^3–7^. This lack of evidence may not necessarily be due to the lack of such ecological processes occurring, but rather because they are both difficult to detect in marine systems and also may have occurred prior to monitoring, especially since most ecological studies are short, providing just a snapshot in time. Long-term ecological monitoring may be required to detect such ecosystem changes.

**Figure 1:**
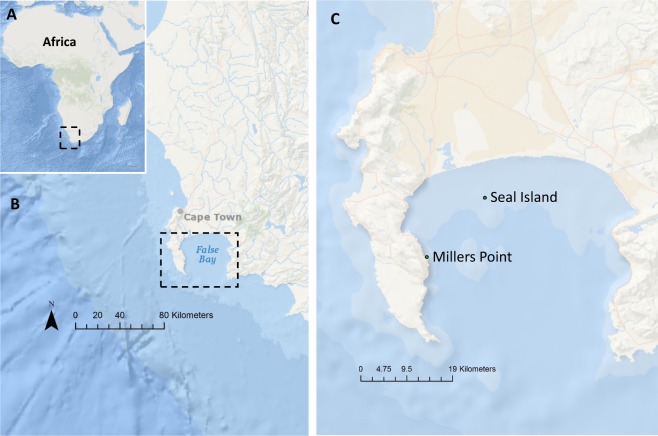
Locations of Seal Island, a Cape fur seal rookery, (**A**) off the Western Cape of South Africa, within (**B**) False Bay. Historically, white sharks actively patrolled the waters of (**C**) Seal Island in the colder months. The only well-known aggregation site for sevengill sharks in False Bay is the inshore kelp beds of Millers Point (**C**), which is ~18 km southwest of Seal Island.

**Figure 2:**
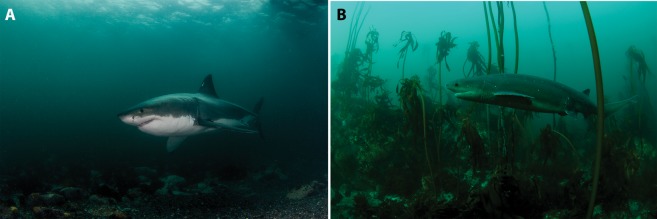
In (**A**), a white shark moves along the rocky, unobstructed bottom, of Seal Island, a Cape fur seal rookery, in central False Bay. In (**B**), a sevengill shark moves through the dense inshore kelp beds off Millers Point, in western False Bay.

Since 2000, we have been monitoring the relative abundance and predatory behavior of white sharks (*Carcharodon carcharias*) at Seal Island, an offshore rookery inhabited by ± 60,000 Cape fur seals (*Arctocephalus pusillus pusillus*), in False Bay, South Africa (Fig. 1). White sharks concentrate their movements at Seal Island during colder months of the year (May-September) to actively predate on Cape fur seals^8^ (Fig. 2A). During warmer months (October-April), white sharks shift the focus of their hunting to inshore areas where they presumably feed on large teleosts and elasmobranchs; however, they are still found around Seal Island, albeit in lower numbers^9,10^. During this time they are often seen scavenging on the carcasses of newborn seal pups that wash off the Island during summer storms.

**Figure 3:**
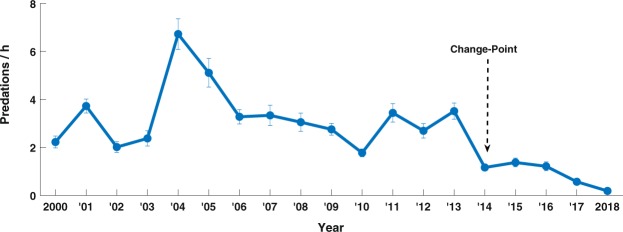
Annual white shark predation rates on Cape fur seals over 18 years of monitoring at Seal Island in False Bay, South Africa. Data are mean ± standard error of white shark predations per hour, averaged across sampling days for each year. A significant change-point in the time-series is indicated with an arrow.

Between 2000 and 2018, a total of 8,076 predatory attacks by white sharks on cape fur seals were recorded during 3,288 hours of standardized observation. Change-point analyses revealed a significant change in annual predation rates by white sharks on Cape fur seals occurring in 2014 (Fig. 3), after which predation rates declined. In 2018, white shark annual predation rates reached an 18-year low of 0.2 ± 0.05 (mean ± standard error) attacks per hour (Fig. 3). The same pattern was found when data from only the colder months (May–September) were analyzed, with a significant change-point also found in 2014 (Supplementary Fig. S1).

**Figure 4:**
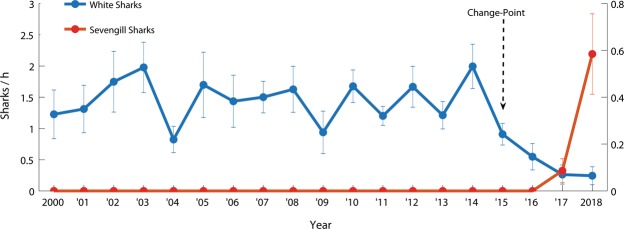
Annual white shark and sevengill shark relative abundance over 18 years of monitoring at Seal Island in False Bay, South Africa. Data are mean ± standard error of white shark sightings per hour (left y-axis) and sevengill shark sightings per hour (right y-axis), averaged across sampling days for each year. A significant change-point in the white shark time-series is indicated with an arrow.

Between 2000 and 2018, a total of 6,333 individual white sharks were sighted during 4,952.4 hours of standardized observational surveys. Change-point analyses revealed a significant change in annual white shark relative abundance patterns occurred in 2015, after which white shark relative abundance declined (Fig. 4). In 2018, white shark relative abundance reached an 18-year low of 0.5 ± 0.1 (mean ± standard error) sightings per hour (Fig. 4). The same pattern was found when data from only the colder months (May–September) were analyzed, with a significant change-point also found in 2015 (Supplementary Fig. S2).

**Figure 5:**
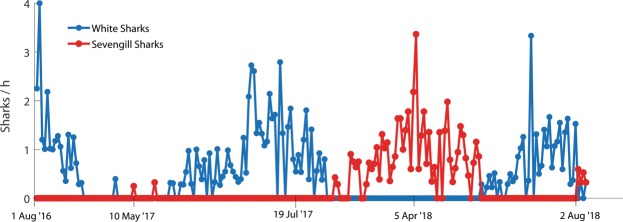
White shark and sevengill relative abundance over last 211 consecutive field days occurring between 1 August 2016 and 4 August 2018. Data are total number of different shark sightings per hour for each sampling day.

In 2017 and 2018, we also recorded for the first time in our surveys, prolonged periods (10+ consecutive sampling days) of complete white shark absence, even during the colder months when white shark abundance and hunting activity had historically peaked (Fig. 5). This included no observations of white sharks over 20 consecutive sampling days that occurred between 27 March and 21 May 2017 and then again over 60 consecutive sampling days that occurred between 3 August 2017 and 29 May 2018 (53 of these sampling days occurred in the cooler months, March through September).

This disappearance of white sharks from our surveys in 2017 coincided with the first recorded observations of sevengill sharks (*Notorynchus cepedianus*) at Seal Island since monitoring began (Fig. 4, Supplementary Fig. S2). Additionally, sevengill shark sightings continued to increase in 2018 as white shark relative abundance further decreased (Fig. 4, Supplementary Fig. S2). Interestingly, sevengill sharks (N = 120 sightings) were only detected during the prolonged periods of complete white shark absence, disappearing from our surveys during the intermittent short periods when white sharks temporarily returned to Seal Island, albeit in very low numbers, only to reappear when the white sharks were again absent from surveys (Fig. 5). On 28 March 2018, at 0945 h, during a period when no white sharks were recorded from our surveys at Seal Island, an estimated 2-m in length sevengill shark rose to the surface and attacked a young-of-the year Cape fur seal. Taken together, these data suggest that the emergence of sevengill sharks at Seal Island was associated with the disappearance of white sharks.

The sevengill shark is widely distributed globally in temperate waters^11^. Detailed dietary studies and stable isotope analyses from sevengill sharks demonstrate this species is an apex predator, feeding primarily on elasmobranchs, large teleosts and seals^12–15^. As concluded by Ebert^14^, it appears that the sevengill shark has no other trophic equivalents in South Africa’s coastal marine ecosystems, with the exception of the white shark. In fact, other than orca whales (*Orcinus orca*), white sharks appear to be the only predator of adult sevengill sharks^13^.

Within False Bay, sevengill sharks frequent inshore kelp beds^16^, with one well-known aggregation site located in the vicinity of Millers Point, located ~18 km southwest of Seal Island (Figs 1 and 2B). However, tracking studies have revealed that white sharks infrequently occur within the areas near this sevengill aggregation site off Millers Point^17^. As suggested by previous research^17^, this may be because the dense kelp obstruct white shark movements and provide structural refugia to their prey, thereby impeding white shark hunting^10,18^. The sevengills aggregating in these kelp beds may thus benefit from both reduced competition for shared prey as well as lowered predation risk from white sharks. Accordingly, we hypothesize that the anomalous emergence of sevengill sharks at Seal Island during 2017 and 2018 was the result of predation- and/or competitive- release in the absence of white sharks. We suggest that the decline in white sharks that began in 2015 likely reached a threshold in 2017 that subsequently led to the emergence of sevengill sharks at Seal Island. It is probable that sevengill sharks may have historically passed by or visited Seal Island prior to the decline in white sharks, but never were bold enough to approach our surface baits in the presence of white sharks and thus were not detected in our surveys. Tracking data from sevengill sharks would be useful for testing these hypotheses.

The reasons for the white shark population declines documented at Seal Island since 2015, combined with prolonged periods of disappearances in our surveys during 2017 and 2018, remain unknown. It is possible these patterns reflect population-level declines for the region, for example, from over-fishing or habitat loss, as suggested from mark-recapture, photo-ID and genetic analysis^19–21^. It is also possible that False Bay’s white sharks have shifted their distributional range elsewhere due to shifts in environmental conditions or prey^22^. While the seal population size in False Bay remains relatively high and stable^10,23^, there could be undocumented declines or distributional shifts in alternative prey needed to sustain this apex predator. Changes in water temperatures associated with climate variability are unlikely to be a driver of the declines as a previous study at Seal Island found no impact of water temperature on inter-annual variations in white shark predation rates^24^. Determining the drivers and scale of the white shark declines documented in this study is a priority area for future research. Further, it remains unknown if and when white sharks may return in historical numbers to Seal Island.

As for any study in the wild, we cannot completely rule out that the unprecedented appearance of sevengill sharks at Seal Island that occurred in 2017 and 2018 could be linked to other factors than white sharks. However, given both species occupy similar trophic positions, with the exception that white sharks are also predators of sevengill sharks, we are unaware of any alterations in prey that would lead to the appearance of sevengill sharks at Seal Island while simultaneously causing the disappearance of white sharks. While water temperature changes could be a factor influencing shark occurrences at Seal Island, our own independent sampling of environmental conditions at Seal Island has found no significant changes in annual mean water temperatures across the 18-year study (Supplementary Fig. S3). Furthermore, previous work at Seal Island found no influence of temperature on inter-annual variations in white shark predation rates^24^. Taken together, while our results are correlative, we are not aware of any major environmental or biological perturbations across the study period that we can attribute to the appearance of sevengill sharks at Seal Island in 2017 and 2018, with the exception of the disappearance of white sharks from our surveys. Strength in our hypothesis also comes from the variability in sightings reported in Fig. 5, where white shark and sevengill shark sightings are inversely related, with alternating absence/presence of white sharks leading to alternating presence/absence of sevengill sharks.

The potential cascading effects from the emergence of sevengill sharks at Seal Island are uncertain. White sharks have been found to affect the abundance, behavior and physiology of Cape fur seals here within the study system^10,25,26^; however, sevengill sharks may not fulfil the same ecological role. Additionally, any behavioral shifts of sevengills away from the inshore kelp beds could also have community-level effects, as have been observed with other kelp-associated predator-prey dynamics (e.g. the classic killer whale–sea otter–urchin–kelp trophic cascade off the Pacific Northwest of the United States^27^.

In summary, our study provided field evidence for ecological changes following the decline and disappearance of an apex predatory shark. Without historical data and time-series monitoring^28^, documenting and disentangling ecological consequences of marine predator declines and recoveries will likely continue to prove challenging.

## Methods

### Study site

Seal Island (34.1374°S, 8.5825°E) is an island rookery for Cape fur seals in False Bay, South Africa (Fig. 1). The underwater topography around the waters of Seal Island features a sharp drop-off along most of the western side of the island, where the water depth drops below 20 m within 50 m of shore, and a broad, shallow shelf along the north east side, where the drop-off occurs only 400 m from shore. The island is inhabited by approximately 60,000 Cape fur seals. The seals typically leave the island in coordinated groups of 5–20 individuals to feed in False Bay or up to 12–30+ km offshore, outside of the Bay, returning to the island at irregular intervals. White sharks actively hunt Cape fur seals at Seal Island during colder months (May through September), with attack rates peaking in June through August^10^. Accordingly, though white sharks occur at Seal Island throughout the year, their residency and relative abundance is higher during colder months^10^.

Year-round, between January 2000 and August 2018, research vessels conducted standardized monitoring of white shark predation events and relative abundance in the waters surrounding Seal Island. Upon daily arrival at the study site, water temperature was recorded using the vessel’s onboard temperature sensor (Furuno model 1870).

### Predation events

Between 07:00 and 09:30 h, sea conditions permitting, predation events by white sharks on Cape fur seals were recorded, following the approach of Fallows *et al*.^29^. As described in Fallows *et al*.^25^, predatory events were detected at the surface by one or more of the following: (1) white shark breach with a seal in its mouth or a seal leaping away from its mouth; (2) a sudden change in the travel behavior of seals, switching from directional porpoising to zigzag evasive maneuvers with a shark in pursuit; (3) a splash accompanied by a blood stain, oil slick, a distinctive odor, and by any of the following indicators: a floating seal head, entrails floating on the surface or trailing from the gill openings of a white shark in the immediate vicinity, and/or highly localized plunge-diving black-backed kelp gulls (*Larus dominicanis vetula*) picking up and feeding on seal entrails. Any subsurface kills could be detected by the appearance of a blood stain at the surface and floating seal entrails.

The duration of each observational period along with the number of predatory attacks by sharks on seals during this period were recorded to calculate white shark predation rates (i.e. number of predation events per hour)^29^.

### Shark relative abundance

After 09:30 h, the research vessel anchored on the southern side of Seal Island and conducted standardized boat-based baited surveys of white sharks following the approach described in Hammerschlag *et al*.^10^. Between 10:00 and 12:00, sea conditions permitting, sharks were attracted to the boat using a large tuna head and a seal decoy. Individual sharks were identified based on a combination of visual makers, including unique scarring, presence/absence of claspers, and individual variation in pigmentation patterns on the gill flaps, pelvic fins, and caudal fins^30^. The duration of each baited survey was recorded, along with the number of different individual sharks observed during this period. Using these data, we calculated the number of different individual sharks observed per hour of baited survey as a metric of relative shark abundance^10^.

### Analyses

To examine for potential annual trends in either white shark relative abundance or predation rates across the 2000–2018 time-series, we calculated the mean number of white sharks sighted per hour as well as the mean number of shark predations per hour, for each year. Change-point statistical analysis was applied to these data to detect if and when any temporal changes may have occurred in each time-series^31^. We also applied change-point analysis to recorded temperature data to test for any temporal changes. Although surveys were conducted year-round, sampling effort was lower in October through January (mean ± s.d 11 ± 7 sampling days per month between February and September compared to 2 ± 2 sampling days per month between October and January). To prevent any potential bias due to reduced survey effort during these summer months, data from October to January were excluded from the time-series change-point analyses. Since white shark residency, abundance and predation rates are highest at Seal Island during colder months, we also conducted a change-point analyses that only included data from cooler months (May through September) for comparison. All change-point analyses were undertaken using Change-Point Analyzer Version 2.3^32^. All other analyses were done using SAS Statistical software.

## Supplementary information

Supplementary Info

## Data Availability

Data reported in this paper are archived in the University of Miami Scholarly Repository.

## References

[1] Estes JA (2011). Trophic downgrading of planet Earth. Science..

[2] Worm B (2013). Global catches, exploitation rates, and rebuilding options for sharks. Mar. Policy..

[3] Baum JK, Worm B (2009). Cascading top-down effects of changing oceanic predator abundances. J.Anim.Ecol..

[4] Ferretti F, Worm B, Britten GL, Heithaus MR, Lotze HK (2010). Patterns and ecosystem consequences of shark declines in the ocean. Ecol. Lett..

[5] Roff G (2016). The ecological role of sharks on coral reefs. Trends. Ecol. Evol..

[6] Grubbs RD (2016). Critical assessment and ramifications of a purported marine trophic cascade. Sci. Rep..

[7] Ruppert JL, Fortin MJ, Meekan MG (2016). The ecological role of sharks on coral reefs: Response to Roff *et al*. Trends. Ecol. Evol..

[8] Martin RA, Hammerschlag N, Collier RS, Fallows C (2005). Predatory behaviour of white sharks (*Carcharodon carcharias*) at Seal Island, South Africa. J. Mar. Biol. Assoc. UK..

[9] Kock A (2013). Residency, habitat use and sexual segregation of white sharks, *Carcharodon carcharias* in False Bay, South Africa. PloS one.

[10] Hammerschlag N (2017). Physiological stress responses to natural variation in predation risk: evidence from white sharks and seals. Ecology.

[11] Compagno, L., Dando, M. & Fowler, S. Broadnose sevengill shark in *Sharks of the world*. 68–69 (Princeton University Press, 2011).

[12] Ebert DA (1991). Observations on the predatory behaviour of the sevengill shark *Notorynchus cepedianus*. S. Afr. J. Marine Sci..

[13] Ebert DA (1991). Diet of the sevengill shark *Notorynchus cepedianus* in the temperate coastal waters of southern Africa. S. Afr. J. Marine Sci..

[14] Abrantes KG, Barnett A (2011). Intrapopulation variations in diet and habitat use in a marine apex predator, the broadnose sevengill shark *Notorynchus cepedianus*. Mar. Ecol.Prog. Ser..

[15] Ebert DA (2002). Ontogenetic changes in the diet of the sevengill shark (*Notorynchus cepedianus*). Mar. Freshwater Res..

[16] Ebert DA (1996). Biology of the sevengill shark Notorynchus cepedianus (Peron, 1807) in the temperate coastal waters of southern Africa. S. Afr. J. Marine Sci..

[17] Kock AA (2018). Summer at the beach: spatio-temporal patterns of white shark occurrence along the inshore areas of False Bay, South Africa. Mov. Ecol..

[18] Wcisel M, O’Riain MJ, de Vos A, Chivell W (2015). The role of refugia in reducing predation risk for Cape fur seals by white sharks. Behav. Ecol. Sociobiol..

[19] Towner AV, Wcisel MA, Reisinger RR, Edwards D, Jewell OJ (2013). Gauging the threat: the first population estimate for white sharks in South Africa using photo identification and automated software. PloS one..

[20] Andreotti S (2016). An integrated mark-recapture and genetic approach to estimate the population size of white sharks in South Africa. Mar Ecol Prog Ser..

[21] Irion DT (2017). Pessimistic assessment of white shark population status in South Africa: comment on Andreotti *et al*. (2016). Mar Ecol Prog Ser..

[22] Hewitt AM, Kock AA, Booth AJ, Griffiths CL (2018). Trends in sightings and population structure of white sharks, *Carcharodon carcharias*, at Seal Island, False Bay, South Africa, and the emigration of subadult female sharks approaching maturity. Environ. Biol. Fish..

[23] Kirkman SP (2013). Spatio‐temporal shifts of the dynamic Cape fur seal population in southern Africa, based on aerial censuses (1972–2009). Mar. Mammal Sci..

[24] Skubel RA, Kirtman BP, Fallows C, Hammerschlag N (2018). Patterns of long-term climate variability and predation rates by a marine apex predator, the white shark *Carcharodon carcharias*. Mar. Ecol. Prog. Ser..

[25] Fallows, C., Martin, R. A., Hammerschlag, N. Comparisons between white shark-pinniped interactions at Seal Island (South Africa) with other sites in California (United States) in *Global Perspectives on the Biology and Life History of the* White Shark (ed. Domeier M. L.) 105–117 (CRC Press, 2012).

[26] De Vos A, O’Riain MJ, Meyer MA, Kotze PGH, Kock AA (2015). Behavior of Cape fur seals (*Arctocephalus pusillus pusillus*) in response to temporal variation in predation risk by white sharks (*Carcharodon carcharias*) around a seal rookery in False Bay, South Africa. Mar. Mammal Sci..

[27] Estes JA, Tinker MT, Williams TM, Doak DF (1998). Killer whale predation on sea otters linking oceanic and nearshore ecosystems. science..

[28] Jackson JB (2001). Historical overfishing and the recent collapse of coastal ecosystems. science..

[29] Fallows C, Fallows M, Hammerschlag N (2016). Effects of lunar phase on predator-prey interactions between white shark (*Carcharodon carcharias*) and Cape fur seals (*Arctocephalus pusillus pusillus*). Environ. Biol. Fish..

[30] Domeier ML, Nasby-Lucas N (2007). Annual re-sightings of photographically identified white sharks (*Carcharodon carcharias*) at an eastern Pacific aggregation site (Guadalupe Island, Mexico). Mar. Biol..

[31] Taylor, W. A. Change-point analysis: a powerful new tool for detecting changes, https://variation.com/wp-content/uploads/change-point-analyzer/change-point-analysis-a-powerful-new-tool-for-detecting-changes.pdf (2000).

[32] Taylor, W. A. Change-Point Analyzer 2.3 shareware program, Taylor Enterprises, Libertyville, Illinois, http://www.variation.com/c (2000).

