# Supplementary material for: Disappearance of white sharks leads to the novel emergence of an allopatric apex predator, the sevengill shark
Source: Sci Rep. 2019 Feb 13;9:1908. doi: 10.1038/s41598-018-37576-6 (PMC6374366; doi:10.1038/s41598-018-37576-6)

Supplementary Information:

Title: Disappearance of white sharks leads to the novel emergence of an allopatric apex predator, the sevengill shark

Authors: Neil Hammerschlag[1,2*], Lacey Williams[3], Monique Fallows[3], Chris Fallows[3]

[1]Rosenstiel School of Marine and Atmospheric Science, Department of Marine Ecosystems and Society, University of Miami, Miami, FL, USA.
[2]Leonard and Jayne Abess Center for Ecosystem Science and Policy, University of Miami, Coral Gables, Coral Gables, FL, USA.
[3]Apex Shark Expeditions, Shop 3 Quayside center, Simonstown, Cape Town 7975, South Africa.

*corresponding author, E-mail: nhammerschlag@miami.edu

Supplementary Figure S1. Annual white shark predation rates on Cape fur seals over 18 years of monitoring at Seal Island in False Bay, South Africa. Data are mean ± standard error of white shark predations per hour, averaged across sampling days for each year. Data presented are based on sampling occurring only during cooler months (May-September). A significant change-point in the time-series is indicated with an arrow.

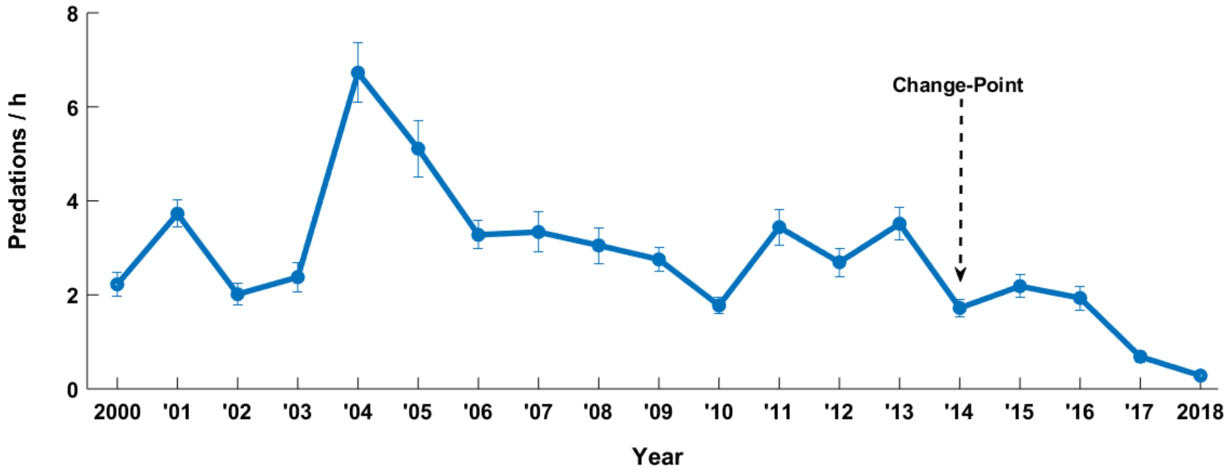

Supplementary Figure S2. Annual white shark and sevengill shark relative abundance over 18 years of monitoring at Seal Island in False Bay, South Africa. Data are mean ± standard error of white shark sightings per hour (left y-axis) and sevengill sharks per hour (right y-axis), averaged across sampling days for each year. Data presented are based on sampling occurring only during cooler months (May-September). A significant change-point in the white shark time-series is indicated with an arrow.

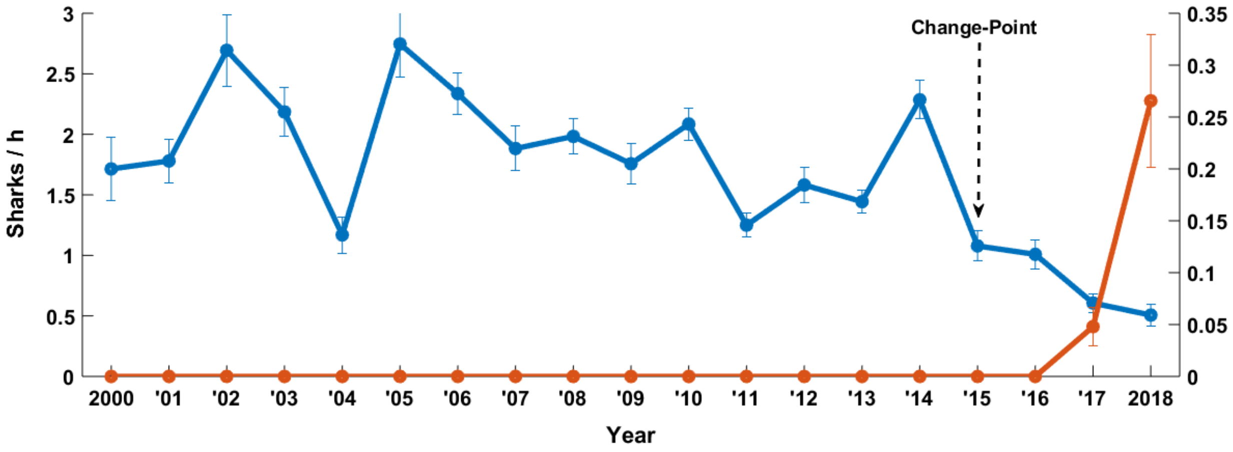

Supplementary Figure S3. Temperatures recorded at Seal Island, in False Bay, South Africa, during 18-years of monitoring. Data are annual means, averaged across sampling days. The green time-series is for temperature recordings from February through September, spanning both warmer and cooler months. The blue time-series is for temperature recordings that occurred only during cooler months, from May through September. Change-point analysis revealed no change-point in either time-series.

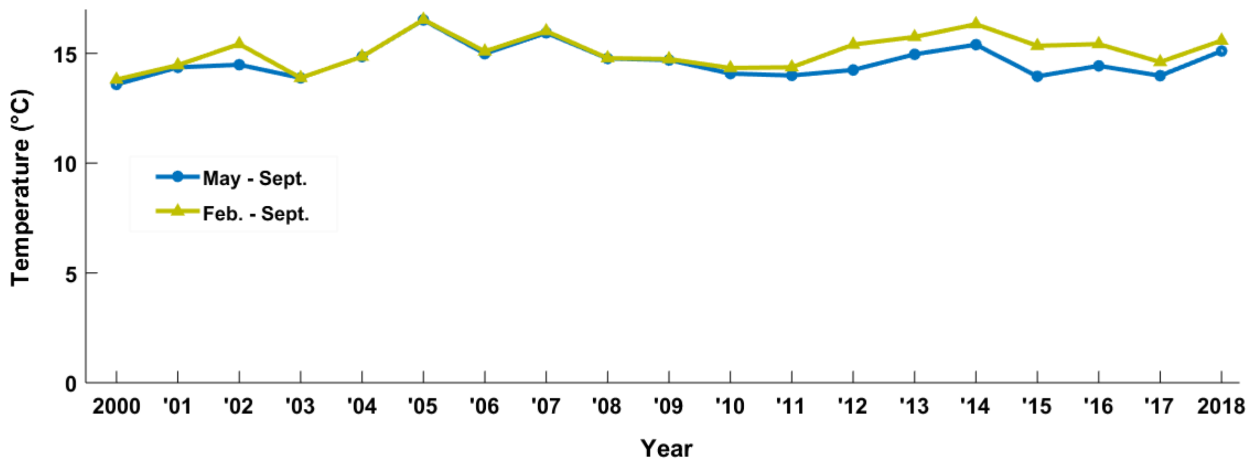

Supplement: Supplementary file 1 — Supplementary Info [file 41598_2018_37576_MOESM1_ESM.pdf]
